# Knowledge platform affordances and knowledge collaboration performance: The mediating effect of user engagement

**DOI:** 10.3389/fpsyg.2022.1041767

**Published:** 2022-12-15

**Authors:** Zeyu Jiao, Chang Li, Jianbin Chen

**Affiliations:** Business College, Beijing Union University, Beijing, China

**Keywords:** knowledge collaboration, social interaction, knowledge platforms, user engagement, knowledge platform affordances, knowledge collaboration performance

## Abstract

Knowledge collaboration is the result of knowledge transfer and social interaction among users on knowledge platforms, and its formation mechanism has attracted much attention. Based on the affordance theory, this paper introduces user engagement as a mediating variable to study the relationship between knowledge platform affordances and knowledge collaboration performance. Data collected from 361 valid questionnaires from the Zhihu platform were analyzed by using SPSS 26.0 and Amos 24.0. The results show that knowledge platform affordances have a direct and positive influence on knowledge collaboration performance as well as an indirect influence through user engagement. Expressly, user engagement undertakes three intermediary paths between knowledge platform affordances and knowledge collaboration performance: knowledge affordances-conscious participation-knowledge collaboration performance, knowledge affordances-enthusiasm-knowledge collaboration performance, and social affordances-social interaction-knowledge collaboration performance. This paper explores the formation process of knowledge collaboration performance by synthesizing affordance and user engagement theories. It clarifies the fundamental role of knowledge affordances in stimulating users’ conscious participation and enthusiasm, and the critical role of social affordances in stimulating social interaction. Therefore, this paper further enriches theories of knowledge collaboration and knowledge platform affordances and provides a practical reference for the strategic optimization and development of knowledge platforms.

## Introduction

Online knowledge communities integrate the functions of knowledge information loading, consulting, retrieval, system management, and instant messaging, which have become essential platforms for knowledge communication and social interactions. At the same time, with the continuous development of knowledge platforms, the role of users has transferred from traditionally passive knowledge receivers to knowledge co-creators. Knowledge platforms such as Quora, Stack Overflow, and Zhihu have all achieved success in knowledge services (Goes et al., 2016). However, some knowledge platforms, such as Baidu Xinzhi and Wukong Q&A, have limited their development due to incomplete functions and low knowledge quality, which eventually lead to the loss of users and the disintegration of the community (Chen, 2021). As an online knowledge platform, the key to survival and development is to increase user traffic and maintain user stickiness. Therefore, attracting potential user groups to join the platform and maintaining active user engagement has become crucial for the sustained development of knowledge platforms.

Knowledge collaboration is the primary operating mechanism of knowledge platforms (Arazy et al., 2013; Kane and Ransbotham, 2016). Based on different needs of knowledge exploration and knowledge contribution, community users interact with each other and promote knowledge innovation to realize the appreciation of knowledge capital and social capital (Chen et al., 2014; Matei et al., 2018). According to Jiao et al. (2021), knowledge collaboration is influenced by knowledge platform affordances in two ways. One is that knowledge affordances provide a resource base for knowledge collaboration and directly affect the knowledge collaboration process. The other is that social affordances provide basic support and public services for knowledge collaboration. Thus, the realization mechanism of knowledge collaboration is not only directly affected by factors such as users’ needs, cognitive conflict, group size, and content quality (Liu F. J. et al., 2020) but also closely related to platform affordances.

How to realize the influencing mechanism of knowledge collaboration performance has become a new challenge.

Enhancing user engagement is a crucial way to expand community traffic. In a large number of studies focusing on platform ecology and value co-creation (Parker and Alstyne, 2005; Zhong et al., 2020), user engagement has received extensive attention as an essential source of value co-creation. It is defined as “a quality of user experience characterized by the depth of an actor’s cognitive, temporal, affective, and behavioral investment when interacting with a digital system” (O'Brien and Cairns, 2016). Meanwhile, from a socio-technical perspective, a knowledge platform is a collection of possibilities and needs of user behaviors of knowledge collaboration in social media and organizational environments (Rice et al., 2017). Platform positioning and organizational characteristics determine its affordances and social resource search and aggregation method (Su et al., 2020). Those affordances could directly determine the user engagement effect. Although there is a complex causal relationship between knowledge platform affordances and user engagement, this paper mainly focuses on the impact of support capabilities on user engagement.

This paper makes a contribution to the literature and practice in lots of ways. In the theoretical aspect, this paper goes deep into the interaction between the platform and users to discuss how to realize the way to improve knowledge collaboration performance and expand the depth of research on affordances and user engagement in the context of knowledge platforms. Existing research has studied the impact of gamification on user engagement in the environment of mobile apps (Ho and Chung, 2020; Bitrián et al., 2021). There is also a study about the relationship between customer engagement and the value creation of company social networks (Zhang et al., 2017). With the improvement of business models, user engagement has become almost a fundamental role of the platform economy. However, most platforms focus heavily on network traffic and user attention, there are still many uncertainties from user access to the platform to forming user engagement. Therefore, the formation mechanism of user engagement has received little attention in current research. In particular, studying user engagement in combination with platform features is a gap in platform user research. Existing research explored the formation mechanism of user engagement in multiple dimensions, focusing on perceived benefits (Wang and Jiang, 2018), trust atmosphere (Yang and Tu, 2018), user value (Ning et al., 2019), and digital touchpoint (Lemon and Verhoef, 2016). Because of the heterogeneity of the platform, the engagement between the platform and the user is bidirectional and it is not enough to study the user engagement without the platform characteristics. The research on this topic is conducive to realizing the integration of platform affordances and user engagement, further enriching the theoretical system of knowledge collaboration mechanism, and realizing theoretical innovation.

In the practical aspect, this paper can provide a decision-making reference for companies to promote user engagement by building platform affordances. The research on this topic can guide more platforms to provide effective affordances and improve user engagement under the premise of unifying user needs and organizational characteristics. In this way, it could help avoid wasting social resources and improve Internet innovation’s success rate.

## Literature review

### Knowledge platform affordances

#### Affordances

Affordance originally refers to the support that objective things can provide for a certain behavior, that is, the possibility of things providing a certain behavior (Gibson, 1986). It refers to the general characteristics of an object which enable it to fulfill the specific needs of individuals. In a certain context, the affordance of something is determined by its actual properties and perceived properties (Norman, 1988). The concept of affordance is emphasized differently in different subject areas. In recent years, it has grown in popularity in organizational research to better understand the impact of a combination of new technologies and organizational characteristics on organizational innovation and functioning (Majchrzak and Faraj, 2013). Especially for social media affordances, they have attracted a lot of attention from researchers as their significant impact on users’ behavior and psychology, and organizations’ communication process. According to Rice et al. (2017), social media affordances refer to the relationship between action possibilities and users’ perceived needs aggregated in social media and the corporate environment under the constraints of the potential features or functions of social media platforms. Based on social media affordances, Majchrzak and Faraj (2013) investigated different ways for employees to engage with the platform and facilitate knowledge sharing. Postigo (2016) analyzed how YouTube can guide users to conduct behaviors that benefit the platform’s commercial interests through the design of its platform architecture. Sun et al. (2019) did research about the influences of corporate social work platforms on employees’ improvisation ability.

Concerning dimensions of affordances, Treem and Leonardi (2012) suggested four dimensions of social media affordances: visibility, associating, editability, and persistence, which have been widely accepted (Fayard and Weeks, 2014; Sun et al., 2019; Zhang, 2020). Affordances of meta-voicing, trigger attending, network-informed associating, and generative role-taking were proposed by Majchrzak and Faraj (2013). Additionally, Rice et al. (2017) proposed six dimensions of functional media affordances. Fox and McEwan (2017) mentioned ten dimensions of communication affordances. Pan and Liu (2017) suggested mobile affordances, which include portability, availability, locatability, and multimediality. The most integrated literature suggested by Karahanna et al. (2018) divided social media affordances into egocentric affordances (self-presentation, content sharing, and interactivity) and allocentric affordances (presence signaling, relationship formation, group management, browsing other’s content, meta-voicing, communication, collaboration, competition, and sourcing). Based on this dimension, the relationship among users’ psychological needs, platform affordances, and platform features is established in the context of social media.

#### Knowledge platform affordances

Knowledge platforms are not only intermediaries in the multilateral market but also heterogeneous production organizations with different value propositions, market orientations, and different resource endowments. It is “productive” (converges and empowers socialized producers) and “knowledgeable” (provides industry-specific knowledge and proprietary resources), and is unique and decisive in the influencing mechanism of knowledge collaboration performance (Chen et al., 2014). Based on the socio-technical perspective, the affordance theory can better demonstrate the interaction between Internet technology, user needs, and organizational characteristics, and better understand the innovation operations under the guidance of affordances. Different from data ability, the value of knowledge platform affordances is not merely created by digital technology. More importantly, it is co-created by the interaction between users, technology, and purposes of use (Ghazawneh and Henfridsson, 2015). By studying the micro-mechanism of the interaction between users and the platform, we can reveal the key elements and institutional arrangements necessary to engage users through the affordances of knowledge platforms, and provide helpful theoretical guidance for the realization of platform value.

The knowledge platform accumulates two types of knowledge resources in the process of supporting the communication between knowledge seekers and contributors: “knowledge about users” and “knowledge produced by users.” The former refers to the knowledge about users’ attributes, social networks, and behaviors accumulated on the platform through digital interaction. It can promote the evolution of collaboration tools, user portraits, and accurate recommendations and is ultimately reflected in platform functions. In this way, it forms the social affordances of the platform (Karahanna et al., 2018; Sun et al., 2019). The latter is the knowledge production results contributed by users and jointly completed, which become the platform’s strategic resources, manifested as the platform’s knowledge affordances (Shi et al., 2020). Based on the social media affordances suggested by Karahanna et al. (2018) and knowledge attributes suggested by Shi et al. (2020), the author suggested 10 affordances of online knowledge platforms by taking Zhihu as an example (Jiao et al., 2021). The result shows that knowledge platforms consist of social affordances and knowledge affordances, which constitute the theoretical basis of knowledge collaboration mechanism in this paper (see Table 1).

**Table 1:** Knowledge platform affordances.

Affordances	Definitions	Example features
*Social affordances*		
Self-presentation	Users can display and present information related to themselves. This includes sharing information that somehow portrays users and shows who they are, their values and preferences, their expertise, etc. Updating descriptive information about themselves, such as gender, occupation and location; and posting content involving pictures and videos related to themselves (Davis et al., 2009; Nardon and Aten, 2012; Halpern and Gibbs, 2013; Junglas et al., 2013)	Posting my own content on Zhihu; updating my profile on Zhihu; writing personal opinions on Zhihu
Content Sharing	Users can share and distribute content unrelated to them to others. (for example, sharing posts, videos, etc.; Kietzmann et al., 2011; Majchrzak and Faraj, 2013; Treem and Leonardi, 2012)	Sharing links of other people’s articles on Zhihu; sharing others’ videos and photos on Zhihu
Relationship Formation	Users can establish relationships with others, including joining groups or online communities (Kietzmann et al., 2011; Treem and Leonardi, 2012)	Following other users on Zhihu; joining an online community (e.g., “Quanzi” on Zhihu)
Browsing Other’s Content	Users can view others’ content and receive alerts to pay attention to others’ content (Davis et al., 2009; Kietzmann et al., 2011; Halpern and Gibbs, 2013)	Browsing others’ content on Zhihu; receiving notifications on Zhihu
Meta-voicing	Users can participate in online conversations by responding to other people’s status, profile, content, and activities online, and viewing other people’s responses to their status, profile, content, and activities. In meta-voice, the user “not only has to express his or her opinion, but also add meta-knowledge to content already online.” (Faraj et al., 2011; Majchrzak et al., 2013)	Voting on a post on Zhihu; commenting on other’s posts on Zhihu; liking what others post on Zhihu
Sourcing	Users are able to ask for resources or funds, including meeting others’ requests for funds or resources (Karahanna et al., 2018)	Asking or answering questions on Zhihu
*Knowledge affordances*		
Reliability	It refers to the extent to which the answers on social Q & A websites make users feel trustworthy and reliable (Zhu et al., 2009). Users think that the answer is of high quality only when they believe that the source and content of the answer are reliable (Kim and Oh, 2009)	The reliability of Zhihu content is reflected in its questions, answers, articles, videos, pictures, etc.
Selectivity	Users can subscribe to specific content or sources of information (Gibbs et al., 2013)	Zhihu involves varied content in multiple sections, and the content within each section is highly subdivided (Cai et al., 2019)
Economies	It means that the subject obtains relatively maximum benefits with relatively minimum investment, so as to obtain benefits most economically and meet the needs of survival and development (Cai, 2008)	Zhihu provides users with a free Q&A community (Zou and Luo, 2017). Users can spend less money asking questions to experts in related fields
Uniqueness	It is defined as individuals pursuing unique characteristics different from others by acquiring, using and disposing of consumer goods (Tian et al., 2001). Novelty is a concept closely related to uniqueness. Novelty refers to the extent to which the answers on social Q & A websites make users feel innovative. Innovative answers will bring new ideas to users and will also be regarded as high-quality answers by users (Zhu et al., 2009; Shah and Pomerantz, 2010)	In-depth content production on Zhihu is different from the knowledge provided by other Q&A platforms. Zhihu online and offline knowledge products are carried out at the same time (Hou and Xiao, 2017)

### User engagement

The concept of engagement has attracted widespread academic attention since Kahn (1900) initial study of the work environment. Therefore, many scholars have conducted related research in various fields. The research includes work by sociologists on social engagement, psychologists on civil engagement, educators on student engagement, and organizational behavior scholars on employee engagement and occupational engagement (Ilic, 2008; Hollebeek, 2011). In recent years, with the development of digital technology, the concept of user engagement in the digital environment has gradually received attention. User engagement is a user experience with aesthetic appeal, interactivity, perceived control, etc. (O’Brien and Toms, 2010), which is more manifested as non-transactional behavior beyond purchase (information sharing, word-of-mouth communication, and value co-creation). It helps companies build valuable connections (Mauda and Kalman, 2016). Some researchers have focused on the user-system properties that provide an engaging experience, which allows researchers to provide guidelines on how to enhance users’ experiences and facilitates the operationalization of user engagement (Jacques, 1996; O’Brien, 2018). In recent decades, the human-computer interaction (HCI) research has increasingly focused on understanding, designing, and measuring user engagement with computers in the health, education, gaming, and news media sectors. Collectively, this research has demonstrated that user engagement is highly context-dependent. Each digital environment has its own set of technological affordances that interact with the motivations of users in order to achieve a particular goal (O’Brien and Cairns, 2016). In 2018, O’Brien et al. define user engagement as “a quality of user experience characterized by the depth of an actor’s cognitive, temporal, affective, and behavioral investment when interacting with a digital system.”

In the marketing domain, Bowden (2009) claimed that customer engagement is a psychological process by which new customers build loyalty and old customers maintain their loyalty to a brand. Similar to user engagement, Van Doorn et al. (2010) put forward that customer engagement is a non-transactional behavior when a customer shows interest in a business or brand for some reasons. Brodie et al. (2013) pointed out that engagement is a multi-dimensional concept that includes cognitive, affective, and behavioral factors, and customers may have different forms of engagement with different stakeholders in different contexts.

Based on the above literature, although it has not reached an agreement about the dimensions of user engagement, it can be seen that most of the explanations emphasize that the relationship between customers or users and enterprises or digital environment contains users’ emotional, cognitive, and behavioral involvement. In the context of online knowledge platforms, users’ experiences are digitally mediated (e.g., online search). Thus, it is timely and vital to understand how individuals interact with these digital environments (O'Brien and Cairns, 2016; O’Brien, 2018). Digital knowledge platforms like Zhihu have two main features. The first one is that Zhihu has social networking functions. Using the sharing function of the platform and the social interactions of users, the platform can promote knowledge sharing and value co-creation. This process needs to rely on interpersonal interaction and knowledge exchange among users, thereby promoting the flow of information on the knowledge platform. Additionally, as a public knowledge question-and-answer platform, Zhihu users may not be brand admirers. Many researchers believe that highly engaged users determine their sustainability on the Zhihu platform. Highly engaged users with a strong passion can provide a vibrant online environment for knowledge platforms. Therefore, this paper will use enthusiasm (emotional element), conscious participation (cognitive element), and social interaction (behavioral element) suggested by Vivek (2009) to measure the user engagement of knowledge platforms.

### Knowledge collaboration performance

Knowledge collaboration was first proposed by Karlenzig et al. (2002) and has been continuously enriched since then. For example, it emphasizes the purpose of integrating complementary knowledge to solve problems (Leijen and Baets, 2002). It also suggests the synergistic effect that the overall benefit is greater than the sum of the individual parts (Leijen and Baets, 2002). In addition, it mentions the transfer of the right information to the right people at the right time, and organizes effective ways to convert knowledge into value (Fan et al., 2007). Tong (2012) comprehensively pointed out that knowledge collaboration is a dynamic process of transferring appropriate information and knowledge to the appropriate target or object at the appropriate time and space, thus realizing knowledge innovation. This is an advanced stage of knowledge management. Chen et al. (2014) further summed up the “appropriateness” of knowledge collaboration at the micro level and the “value-added” effect at the macro level, and proposed two crucial dimensions to measure the performance of knowledge collaboration: knowledge capital appreciation and social capital appreciation. This is also the research basis of this paper.

The knowledge collaboration performance of the knowledge platform is the ultimate value realization method of knowledge (Chen et al., 2014). At present, there is no unified definition in the academic community. However, the conclusion that it includes two dimensions of knowledge capital appreciation and social capital appreciation has been recognized by many scholars (Chen et al., 2014; Strickland, 2014; Bharati et al., 2015; Seo, 2020; Zhou et al., 2022). Social capital in a virtual community represents the connection between people and the personal wealth accumulated through the connection, which is the trust cooperation, and collective behavior established in the interpersonal network of the community (Chang and Chuang, 2011). The social capital theory believes that the network of relationships embodied by individuals has an impact on interpersonal knowledge-sharing behavior (Nahapiet and Ghoshal, 1998). In its simplest form, social capital is what an individual knows about someone that extends what you have (economic capital) or know (human capital). A basic assumption about social capital is that social systems have immediate or expected value. The success of viral marketing, open-source communities, and social media makes the purpose of social capital very attractive (Han et al., 2020). Therefore, gaining social capital appreciation has also become one of the main purposes for users to participate in knowledge collaboration. Social capital includes three dimensions: structural dimension, relationship dimension, and cognitive dimension (Seo, 2020). The structural dimension measures the social connection status, that is, the relationship between members of the knowledge platform. The relationship dimension is the strength of the relationship between members, which is reflected in the individual’s sense of trust, identity, and reciprocity with other users in the knowledge platform. That is, when an individual gets help from others, he will give each other in return (Chow and Chan, 2008). The cognitive dimension is mainly reflected in the shared vision of the members of the knowledge platform, that is, the members’ common interests, viewpoints, and values (Zhao et al. Hair et al., 2010).

Compared with the appreciation of social capital, the appreciation of knowledge capital is more direct (Chen et al., 2014), which is directly reflected in the acquisition of knowledge by users. Due to the sharing and non-attrition of knowledge capital, the appreciation of knowledge capital is not only manifested in the increase of explicit knowledge (such as experience summary, process documentation, and knowledge base) or the final explicit knowledge product delivered to customers. The implicit knowledge achievements also become the value-added part of knowledge capital, manifested as the improvement of individual and team capabilities, the accumulation of experience, and the improvement of processes (*ibid*). The value-added of explicit knowledge capital mainly measures the knowledge achievements ultimately formed by knowledge collaboration and jointly owned by organizations or teams, such as patents, processes, and regulations, as well as the increase of knowledge units such as program library, rule base, knowledge base, and case base. The appreciation of implicit knowledge capital mainly measures the increase of individual experience and skills, the improvement of team ability, and the improvement of organizational culture and practice (Hedlund and Nonaka, 1993).

## Research models and hypotheses

### Knowledge platform affordances and knowledge collaboration performance

Previous research has shown that users’ psychological needs drive them to participate in the use of knowledge platforms, and the affordances provided by the organizational features of knowledge platforms can be used to meet such psychological needs (Karahanna et al., 2018; Jiao et al., 2021). Therefore, platform affordances comprehensively reflect organizational features and customer needs. Among them, social affordances are the product of the interaction between users’ psychological needs and platform organizational features, while knowledge affordances are the interaction product of platform knowledge resources and users’ psychological needs (Shi et al., 2020). It is easy to conclude that knowledge platform affordances offer two possibilities for user behavior. On the one hand, knowledge affordances allow users to increase new knowledge by providing a resource base for knowledge collaboration, so as to facilitate knowledge capital appreciation. On the other hand, social affordances can encourage users to socialize through its social functions, so as to influence social capital appreciation. Thus, H1 and H2 are suggested.

*H1:* Knowledge affordances have a positive influence on knowledge collaboration performance.

*H2:* Social affordances have a positive influence on knowledge collaboration performance.

### The mediating effect of user engagement

#### Knowledge platform affordances and user engagement

Based on the above analysis, knowledge platform affordances are divided into social affordances and knowledge affordances. Social affordances include self-presentation, content sharing, relationship formation, browsing other’s content, sourcing and meta-voicing， and knowledge affordances refer to knowledge attributes. The psychological needs of users promote the use of knowledge platforms to a certain extent. At the same time, knowledge platforms also provide affordances to meet user needs. From a socio-technical perspective, a knowledge platform is a collection of relationships that aggregate user behavior possibilities and needs in social media and organizational environments (Rice et al., 2017). Its value is not only created by digital technology but co-created by the interaction between users, technology, and purpose of use (Ghazawneh and Henfridsson, 2015). Ongus and Nyamboga (2019) also mentioned that the quality and relevance of technical resources and information content are closely related to user needs. Therefore, the platform characteristics formed under the support of technology and resources enable the realization of knowledge platform affordances. Over the past two decades, the Human-Computer Interaction (HCI) community has become gradually interested in understanding, designing, and measuring user engagement in numerous computer-mediated health, education, gaming, social and news media, and search applications (O’Brien and Cairns, 2016). Overall, this work shows that user engagement is highly context-dependent: each digital environment has unique technological and social affordances that interact with users’ motivations to achieve some desired purpose.

Vivek (2009) proposed three dimensions of user engagement: enthusiasm, conscious participation, and social interaction. In this scale, conscious participation is the reflection of the cognitive element in user engagement. Enthusiasm is the emotional factor, and social interaction is the behavioral factor. From users’ perspectives, user engagement may come from the fact that their needs are met during the process of their engagement or use of digital platforms (Gummerus et al., 2012). According to the existing literature, conscious participation means users’ intentional participation in activities and they have some cognition with the activities (Vivek, 2009). In marketing activities, reason-oriented customers desire quick and easy access to information about the use of services and products (Zhang et al., 2017). It is common for society-oriented customers to engage in conversation and interaction with others who share similar interests or needs when shopping (Babin et al., 1994). A meaningful connection between people can be created by user engagement (Luthans and Peterson, 2002). Enthusiasm refers to users’ engagement with intense excitement or passion (Vivek, 2009). In the literature of work engagement and customer engagement, enthusiasm is considered to be a positive emotion. It is characterized by a high level of excitement, which is an active and lasting emotion (Bloch, 1986). Vivek (2009) also stressed the importance of enthusiasm for capturing users’ strong excitement and focus. In terms of social interaction, it means the interaction and communication of ideas, feelings, and opinions among users (Vivek, 2009). Through social interaction, consumers can quickly and easily obtain information about relevant products, thereby building intimacy with like-minded people (Muniz and O’guinn, 2001). In the context of knowledge platforms, knowledge platforms with different affordances could facilitate user engagement in different ways. For example, in the context of Zhihu, people who use knowledge platforms with knowledge affordances may often browse content on Zhihu (conscious participation), spend much time on Zhihu to learn knowledge (enthusiasm), and exchange ideas in Zhihu communities (social interaction). Thus, the following hypotheses are proposed:

*H3:* Knowledge affordances have a positive influence on conscious participation.

*H4:* Knowledge affordances have a positive influence on enthusiasm.

*H5:* Knowledge affordances have a positive influence on social interaction.

Additionally, people who use platforms with social affordances are likely to pay lots of attention to Zhihu communities (conscious participation), be passionate about Zhihu (enthusiasm), and enjoy interacting with other users (social interaction). Therefore, digital environments attract users for different reasons (e.g., to learn knowledge, to socialize with people) with different affordances (knowledge affordances and social affordances) to promote engagement. Therefore, hypotheses are suggested as follows:

*H6:* Social affordances have a positive influence on conscious participation.

*H7:* Social affordances have a positive influence on enthusiasm.

*H8:* Social affordances have a positive influence on social interaction.

#### User engagement and knowledge collaboration performance

Knowledge collaboration is an effective way to convert knowledge into value and is the core business process of the platform system (Chen et al., 2014). The impact of user engagement on knowledge collaboration could be manifested in conscious participation, enthusiasm, and social interaction. In terms of conscious participation, users’ cognitive and conscious participation in knowledge platforms may enhance knowledge collaboration performance. Because in knowledge platforms, some users who are reason-oriented may want to have a quick and comprehensive understanding of useful content (such as searching answers for their questions). Some society-oriented users would like to communicate with those who possess the same interests, goals, or needs (Babin et al., 1994). Thus, individuals with different cognition participating in knowledge platforms may obtain such values as acquiring knowledge or skills (knowledge capital appreciation), as well as building their social circles (social capital appreciation; Jaakkola and Alexander, 2014).

With regard to enthusiasm, Glassman and McAfee (1990) suggest that users with enthusiasm are more likely to take risks, which allows them to be willing to take the initiative to reduce misunderstandings and avoid uncertainty. In the context of knowledge platforms, enthusiastic users are more likely to alleviate anxiety and uncertainty, so they could increase trust in the content of knowledge platforms. On the basis of this, interactions allow users to get the needed knowledge and information (Lanier and Hampton, 2008), which could improve knowledge capital appreciation. In addition, this enables the users to present and express themselves in a way they like, so as to increase social capital appreciation (Kaplan and Haenlein, 2010; Gummerus et al., 2012).

It is well-documented that social interaction has a significant impact on value co-creation (Zhang et al., 2017). This kind of value is created by forming mutual trust (Stewart and Pavlou, 2002). Knowledge platforms like Zhihu, with a higher level of interactivity, can attract users to discuss content and respond to questions quickly (Teeni, 2001). For example, the function of the online community enables people who have similar interests to discuss topics they are interested in together. As a result, users can get information and learn knowledge in a quick and easy way, thereby facilitating individuals to know each other and become friends (Muniz and O’guinn, 2001). Building closer relationship can improve both knowledge and social capital appreciation. Therefore, we propose:

*H9:* Conscious participation has a positive influence on knowledge collaboration performance.

*H10:* Enthusiasm has a positive influence on knowledge collaboration performance.

*H11:* Social interaction has a positive influence on knowledge collaboration performance.

#### The mediating effect of user engagement

Based on the former research, it could be seen that platform provides function affordances and resource affordances according to mission and positioning. It helps users realize the aggregation of psychological needs with technology and products, while user engagement is from the fact that users’ needs are met during the process of using platforms (O’Brien and Cairns, 2016). As a result, it is more likely that knowledge platform affordances can promote user engagement of conscious participation, enthusiasm, and social interaction. Therefore, knowledge platform affordances constitute a prerequisite for user engagement.

From a social-technical perspective, online knowledge platforms are composed of the behavioral possibilities and needs of users in social media and organizational settings (Rice et al., 2017). Based on the literature, the purpose of social media affordances is to trigger user engagement, such as sharing of information and interaction, through social media sites (Majchrzak et al., 2013; Cabiddu et al., 2014). In this way, users are enabled to acquire resources and create values (Lin and Kishore, 2021). In the context of knowledge platforms, social affordances facilitate users to use the social functions of Zhihu. People who have a demand for socializing will be highly engaged in Zhihu. Highly engaged users are more willing to expand their social networks and share knowledge on Zhihu to find those who share the same interests, goals, or needs and then communicate with them, which will facilitate users’ social interaction on Zhihu. From this process, it could bring knowledge capital increase and social capital increase. It is the same reasoning that can be applied to knowledge affordances. Therefore, the following hypotheses are suggested.

*H12:* User engagement plays the mediating role between knowledge platform affordances and knowledge collaboration performance.

The sub-hypotheses of this hypothesis are as follows:

*H12a:* Conscious participation mediates the relation between knowledge affordances and knowledge collaboration performance.

*H12b:* Enthusiasm meditates the relation between knowledge affordances and knowledge collaboration performance.

*H12c:* Social interaction mediates the relation between knowledge affordances and knowledge collaboration performance.

*H12d:* Conscious participation mediates the relation between social affordances and knowledge collaboration performance.

*H12e:* Enthusiasm meditates the relation between social affordances and knowledge collaboration performance.

*H12f:* Social interaction mediates the relation between social affordances and knowledge collaboration performance.

**Figure 1:**
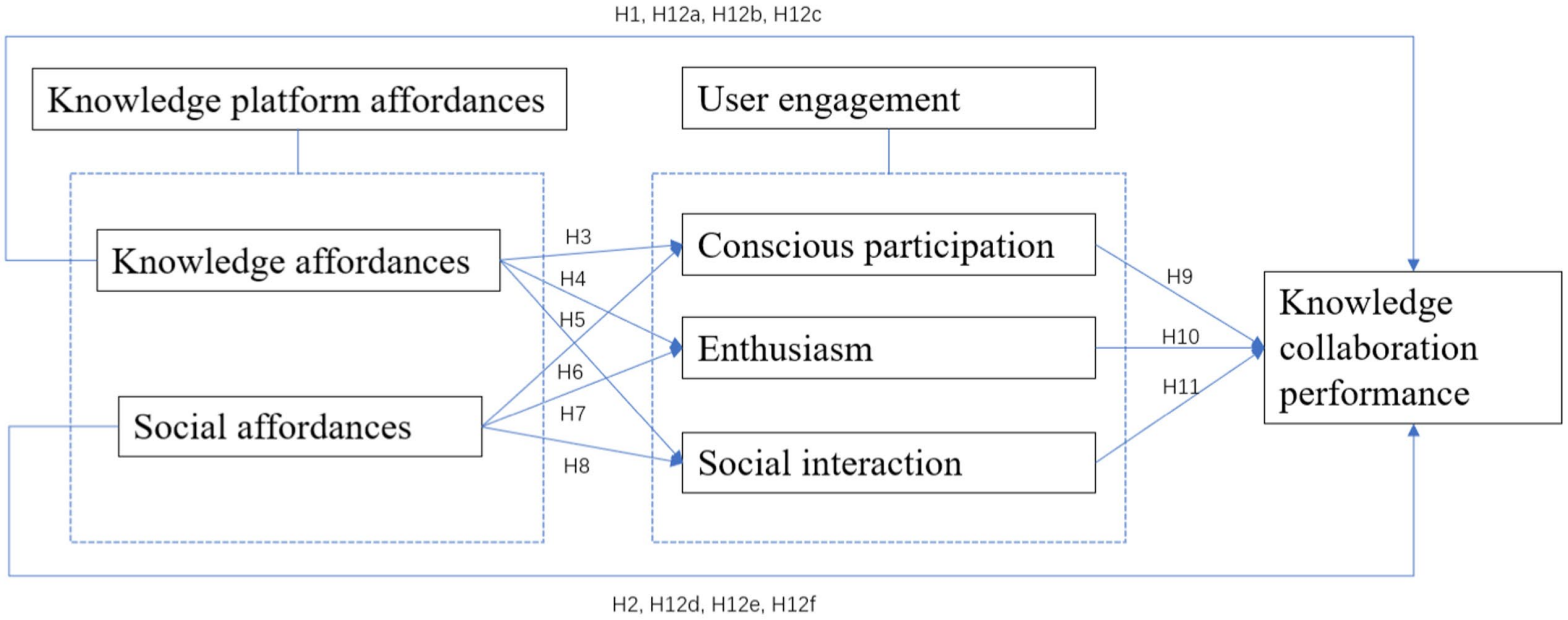
The conceptual model.

A conceptual framework is established based on the theoretical analysis above, as shown in Figure 1. This model has illustrated the influence mechanism of different dimensions of user engagement on knowledge collaboration performance. That is, knowledge platform affordances not only have a direct influence on knowledge collaboration performance, but also exert an indirect influence through user engagement.

## Research methods

### Research process

On the basis of existing research and related theoretical viewpoints, this paper has established an overall theoretical framework in the previous chapter and clearly stated the relationship between each variable. However, establishing the theoretical framework also requires relatively scientific and reasonable empirical research methods to test it to clarify further the relationship between the variables in the framework, which inevitably involves the design of the entire research process.

This paper selects the Zhihu application as the representative of knowledge platforms for research. As one of the earliest socialized knowledge communities in China, Zhihu has grown steadily since its launch in January 2011, and the knowledge payment system has gradually matured. In 2018, Zhihu’s official data showed that the number of users exceeded 220 million. According to data from iResearch, from August 2019 to July 2020, the monthly independent numbers of Zhihu mobile terminals were stable between 46 million and 56 million (Guo, 2020). Due to Zhihu’s rich experience in operating knowledge communities, it has always maintained strong competitiveness. Since this paper focuses on the impact of knowledge platform affordances and user engagement on knowledge collaboration performance, which makes it challenging to measure related variables through open data from Zhihu, this paper adopts the questionnaire survey method to collect relevant data. Furthermore, the overall data type of this paper belongs to cross-sectional data, and all variable data are obtained through questionnaires. The scales in the questionnaire refer to the mature scales of existing research, and the questionnaire is designed in combination with the Zhihu platform. Then, a professional data collection company is used to issue questionnaires. Then, data collection, input, and analysis are carried out to empirically test the relationship between knowledge platform affordances, user engagement, and knowledge collaboration performance.

### Questionnaire design

The design of the questionnaire directly affects whether the obtained data fit the research needs. It is a prerequisite for obtaining valid and reliable data. Therefore, the questionnaire design is the first work to be done before the start of the questionnaire survey. On the basis of existing research, this paper adopts the following steps to design the questionnaire. First, studying relevant literature. Second, engaging in the dialog and discussion with academic experts, and communicating with some Zhihu users on the question design in the questionnaire. Finally, conducting a pre-test and completing the final draft of the questionnaire on this basis (see questionnaires in Appendix 1). Based on the literature, the primary measurement scales of each variable are shown in Table 2.

**Table 2:** Main measurement scales in the research model.

Variable	Measurement scales	Sources
Knowledge platform affordances	Social affordances	Postigo (2016), Rice et al. (2017), Dong (2018), Karahanna et al. (2018), Shi et al. (2020)
	Knowledge affordances	
User engagement	Conscious participation	Vivek (2009), O’Brien and Cairns (2016), and O’Brien (2018)
	Enthusiasm	
	Social interaction	
Knowledge collaboration performance	Knowledge capital appreciation	Chow and Chan (2008), Zhao et al. (2010), Chang and Chuang (2011), Chen et al. (2014)
	Social capital appreciation	

### Data collection

According to the report, Zhihu users are mainly from developed cities, and the proportion of users in first-and second-tier cities is as high as 75.4% (Guo, 2020). Therefore, the questionnaire sample mainly selects users from first-and second-tier cities in China. Considering that our research objects need to meet certain conditions,

for example, they must be people who have used Zhihu and have a certain understanding of its functions, we hired a professional questionnaire survey company to distribute 500 online questionnaires to long-term users of Zhihu. Additionally, the survey company gave small gifts as rewards to users who responded to the questionnaire, so as to improve the recovery rate and effectiveness of the questionnaire. In order to ensure the authenticity of the questionnaire as much as possible, it is emphasized that the purpose of this answer is to collect relevant data for scientific research, and there are no other commercial considerations. It also shows that there is no problem of revealing key information, so users are required to fill in the answer according to the actual situation. Moreover, the questionnaires are filled out anonymously, and the relevant information of the respondents will not be disclosed. Our investigation started in April of 2022 and ended in May of the same year. Three hundred sixty-one valid questionnaires were obtained in the final.

It should be noted that since each questionnaire used in this paper is completed by the same person, it is necessary to pay attention to the problem of common method variance (MacKenzie and Podsakoff, 2012). For this problem, this paper adopts two methods of program control and statistical method control to solve it. First of all, from the above, in the research process, the author thoroughly emphasized that there is no right or wrong answer information, and it is used for research rather than commercial purposes. In addition, for the respondents, they answered the questions anonymously to reduce the bias of answering tendency. Secondly, the author also performed the Harman single-factor test on the recovered data and put all the items together for exploratory factor analysis. The results showed that the factor loading of the first principal component was 27.216%, and it can be seen that the common method variance is not obvious. Therefore, this paper believes that the sample data used does not have serious common method variance problems.

## Results and analysis

This study used SPSS 26.0 and Amos 24.0 statistical software to analyze the research samples. Among them, the analysis methods involved in SPSS 26.0 in this study include descriptive statistics, reliability analysis, and exploratory factor analysis, while methods involved in AMOS 24.0 include confirmatory factor analysis and structural equation model analysis.

### Descriptive statistics analysis of samples

The survey finally collected 361 valid questionnaires. The basic information about the samples is shown in Table 3.

**Table 3:** Descriptive statistics.

Variable	Types	FREQ	PCT
The number of days of using Zhihu in the last month	5–10 days	57	15.8
	10–15 days	188	52.1
	15–20 days	71	19.7
	More than 20 days	45	12.5
Gender	Male	244	67.6
	Female	117	32.4
Age	Under 20 years old	27	7.5
	20–30 years old	140	38.8
	30–40 years old	79	21.9
	40–50 years old	69	19.1
	Over 50 years old	46	12.7
Education level	Colleges	67	18.6
	Bachelor	167	46.3
	Master	65	18
	Doctor	62	17.2
Occupation	Students	47	13
	Researchers	51	14.1
	Managers	47	13
	Technical (or R&D) personnel	63	17.5
	Business people	50	13.9
	Freelancers	58	16.1
	Other	45	12.5
The number of years you have used Zhihu	Within 1 year	25	6.9
	1–3 years	59	16.3
	3–5 years	150	41.6
	5–7 years	72	19.9
	7 years and above	55	15.2

### Exploratory factor analysis

In this paper, SPSS 26.0 software was used to conduct exploratory factor analysis to test the construct validity of the scale. First, through the Kaiser-Meyer-Olkin (KMO) test and Bartlett’s sphere test to see if the data can be subjected to factor analysis. According to Kaiser (1974), KMO above 0.90 indicates that the scale is very suitable for factor analysis. If the KMO is between 0.8 and 0.9, it is suitable for factor analysis. If the KMO is below 0.5, it is very unsuitable for factor analysis. In addition, factor analysis can be done when the statistical significance probability of Bartlett’s Test of Sphericity is less than or equal to the significance level.

Through repeated exploratory factor analysis, PA4 and UE2 have cross-loadings, and UE12 factor loading is lower than 0.4. After excluding items that do not meet the requirements, 24 items are finally retained.

The Bartlett sphericity test shows that Bartlett *χ*^2^ = 3679.161 and *p* < 0.001, which are obtained by performing the test of the correlation matrix on the questionnaire. It indicates that there are common factors among the 24 items of the questionnaire, and it is necessary to carry out the factor analysis on this correlation matrix. At the same time, the KMO measure of sampling adequacy is calculated, and the result shows KMO = 0.862, indicating that it is suitable for factor analysis.

It can be seen from the table below that the factor is intercepted based on the characteristic root >1. After factor extraction is performed on 24 items, six factors are finally extracted, and the six factors cumulatively explain 65.786% of the total variation, which can explain most of the variance. The first factor represents SA (Social affordances) with factor loadings ranging from 0.782 to 0.727; the second factor represents KCP (Knowledge collaboration performance) with factor loadings ranging from 0.782 to 0.664; the third factor represents EN (Enthusiasm) with factor loadings ranging from 0.848 to 0.685; the fourth factor represents SI (Social interaction) with factor loadings ranging from 0.851 to 0.776; the fifth factor represents CP (Conscious participation), with factor loadings ranging from 0.796 to 0.771; the sixth factor represents KA (Knowledge affordances), and factor loadings ranged from 0.799 to 0.767. The loadings of the six factors are all >0.5, and the item distribution after factor rotation is consistent with the theoretical hypotheses of the questionnaire structure, so the revised questionnaire has good construct validity (Table 4).

**Table 4:** Factor analysis results.

	1	2	3	4	5	6
PA6	**0.782**	0.110	−0.059	0.016	0.074	0.056
PA10	**0.779**	0.117	0.089	0.093	0.005	−0.015
PA5	**0.774**	0.091	−0.025	0.028	0.033	0.178
PA7	**0.768**	0.107	−0.031	0.103	−0.033	−0.002
PA9	**0.733**	0.064	0.103	0.107	−0.030	0.134
PA8	**0.727**	0.147	0.089	0.117	0.052	0.066
KCP5	0.191	**0.782**	0.172	0.119	0.106	0.094
KCP4	0.129	**0.765**	0.201	0.129	0.128	0.115
KCP3	0.156	**0.744**	0.180	0.085	0.126	0.086
KCP2	0.071	**0.677**	0.259	0.022	0.211	0.060
KCP1	0.161	**0.664**	0.147	0.203	0.124	0.189
UE8	0.057	0.181	**0.848**	0.095	0.075	0.000
UE6	0.048	0.241	**0.818**	0.083	0.109	0.001
UE7	0.001	0.256	**0.712**	0.046	0.098	0.113
UE5	0.023	0.147	**0.685**	0.139	0.199	0.156
UE10	0.139	0.128	0.121	**0.851**	0.046	−0.079
UE11	0.085	0.082	0.151	**0.798**	0.066	0.057
UE9	0.161	0.192	0.035	**0.776**	0.062	−0.001
UE4	−0.008	0.170	0.170	0.122	**0.796**	0.113
UE3	0.006	0.112	0.173	0.071	**0.784**	0.035
UE1	0.065	0.251	0.072	−0.011	**0.771**	0.096
PA1	0.158	0.152	0.089	−0.061	−0.010	**0.799**
PA3	0.177	0.107	0.106	0.003	0.130	**0.795**
PA2	0.004	0.121	0.028	0.036	0.109	**0.767**
Characteristic root	3.700	3.111	2.711	2.149	2.064	2.053
% of Variance	15.417	12.964	11.297	8.955	8.599	8.554
Cumulative %	15.417	28.380	39.677	48.632	57.232	65.786

PA, knowledge platform affordances; KCP, knowledge collaboration performance; UE, user engagement. The bold values represent factor loadings greater than 0.5.

### Confirmatory factor analysis

This paper used AMOS 24.0 software to conduct confirmatory factor analysis on the samples. Confirmatory factor analysis is a statistical analysis of social survey data. Confirmatory factor analysis explores whether the factor structure model of the scale fits the actual data collected, and whether the indicator variables can be effectively used as the procedures for measuring latent variables. In this study, the maximum likelihood method was used to estimate the model, and the fit of the model was verified by the following indicators: (1) Chi-square (*χ*^2^) test. The *χ*^2^ index is the most basic test index for model fitting, and the *χ*^2^/*df* value is generally used to test. The smaller the value, the higher the simulation fit. Usually, when *χ*^2^/*df* < 3, it means that the model has a good fit (Huang, 2004). (2) The root mean square error of approximation (RMSEA). It is sensitive to the wrong model and is an ideal fitting indicator. The closer the value of RMSEA is to 0, the better the model fit. Usually when RMSEA <0.08, the model has a good degree of fit (Browne and Cudeck, 1993). (3) Standardized root mean square residual (SRMR). Its value ranges from 0 to 1. When SRMR <0.08, it indicates that the model fits well (Hu and Bentler, 1999). (4) Comparative fit index (CFI), Tucker-Lewis index (TLl), and incremental fit index (IFI). Usually, CFI > 0.9, TLI > 0.9, and IFI > 0.9 indicate a good model fit (Wu, 2013). Table 5 shows that the ideal value is reached, thus indicating that the confirmatory factor analysis model fits well.

**Table 5:** Confirmatory factor analysis.

Measure	*χ* ^2^	*df*	*χ*^2^/*df*	RMSEA	SRMR	CFI	TLI	IFI
Threshold			<3.00	<0.08	<0.08	>0.9	>0.9	>0.9
Results	388.828	237	1.641	0.042	0.043	0.957	0.949	0.957

From Table 6, it can be seen that the factor loadings of KA, SA, CP, EN, SI, and KCP are all above 0.5. The CR values are all above 0.7, and the AVE values are all above 0.5. According to Hair et al., (2010)’s suggestion in validity evaluation, the absolute value of factor loading should be at least 0.5, and the best index value should be above 0.7. The average variance extraction (AVE) index value should be above 0.5, and the reliability index value should be higher than 0.7. Therefore, this questionnaire has good convergent validity.

**Table 6:** Convergent validity.

Constructs	Items	Factor loading	*t*-value	CR	AVE
KA	PA1	0.737	–	0.752	0.505
	PA2	0.603	9.533		
	PA3	0.780	10.569		
SA	PA5	0.746	–	0.868	0.524
	PA6	0.738	13.412		
	PA7	0.718	13.037		
	PA8	0.705	12.810		
	PA9	0.695	12.610		
	PA10	0.737	13.397		
CP	UE1	0.703	–	0.768	0.527
	UE3	0.660	10.375		
	UE4	0.807	11.202		
EN	UE5	0.652	–	0.836	0.564
	UE6	0.825	12.524		
	UE7	0.686	10.963		
	UE8	0.823	12.508		
SI	UE9	0.709	–	0.799	0.573
	UE10	0.865	12.144		
	UE11	0.684	11.363		
KCP	KCP1	0.676	–	0.855	0.543
	KCP2	0.662	11.134		
	KCP3	0.721	11.998		
	KCP4	0.800	13.078		
	KCP5	0.812	13.217		

KA, knowledge affordances; SA, social affordances; CP, conscious participation; EN, enthusiasm; SI, social interaction; KCP, knowledge collaboration performance.

The statistics in Table 7 show the correlation coefficient between each variable and the square root of AVE. According to the method proposed by Fornell and Larcker (1981), whether the square root of AVE is higher than the correlation coefficient between two variables is used to judge the discriminant validity. Based on the data, the questionnaire shows good discriminant validity.

**Table 7:** Discriminant validity.

Constructs	KA	SA	CP	EN	SI	KCP
KA	**0.711**					
SA	0.322***	**0.724**				
CP	0.309***	0.112	**0.726**			
EN	0.246***	0.148*	0.432***	**0.751**		
SI	0.032	0.310***	0.237***	0.320***	**0.757**	
KCP	0.405***	0.393***	0.510***	0.582***	0.394***	**0.737**

*p < 0.05; **p < 0.01; ***p < 0.001. The diagonal elements represent the square root of the AVE. The bold values represent the square root of the AVE.

### Reliability analysis

Reliability refers to the consistency or stability of measurement results obtained according to measurement tools. In this paper, Cronbach’s Alpha is used to test the internal consistency reliability of the questionnaire. Devellis (1991) put forward that the value of the α coefficient between 0.65 and 0.70 is the minimum acceptable value.

In this paper, the reliability coefficient value of every variable is above 0.7, so the reliability of the questionnaire is good (see Table 8).

**Table 8:** Reliability analysis.

Constructs	Cronbach’s alpha	Number of items
KA	0.746	3
SA	0.868	6
CP	0.763	3
EN	0.832	4
SI	0.791	3
KCP	0.854	5

### Structural equation modeling for the main effects

**Figure 2:**
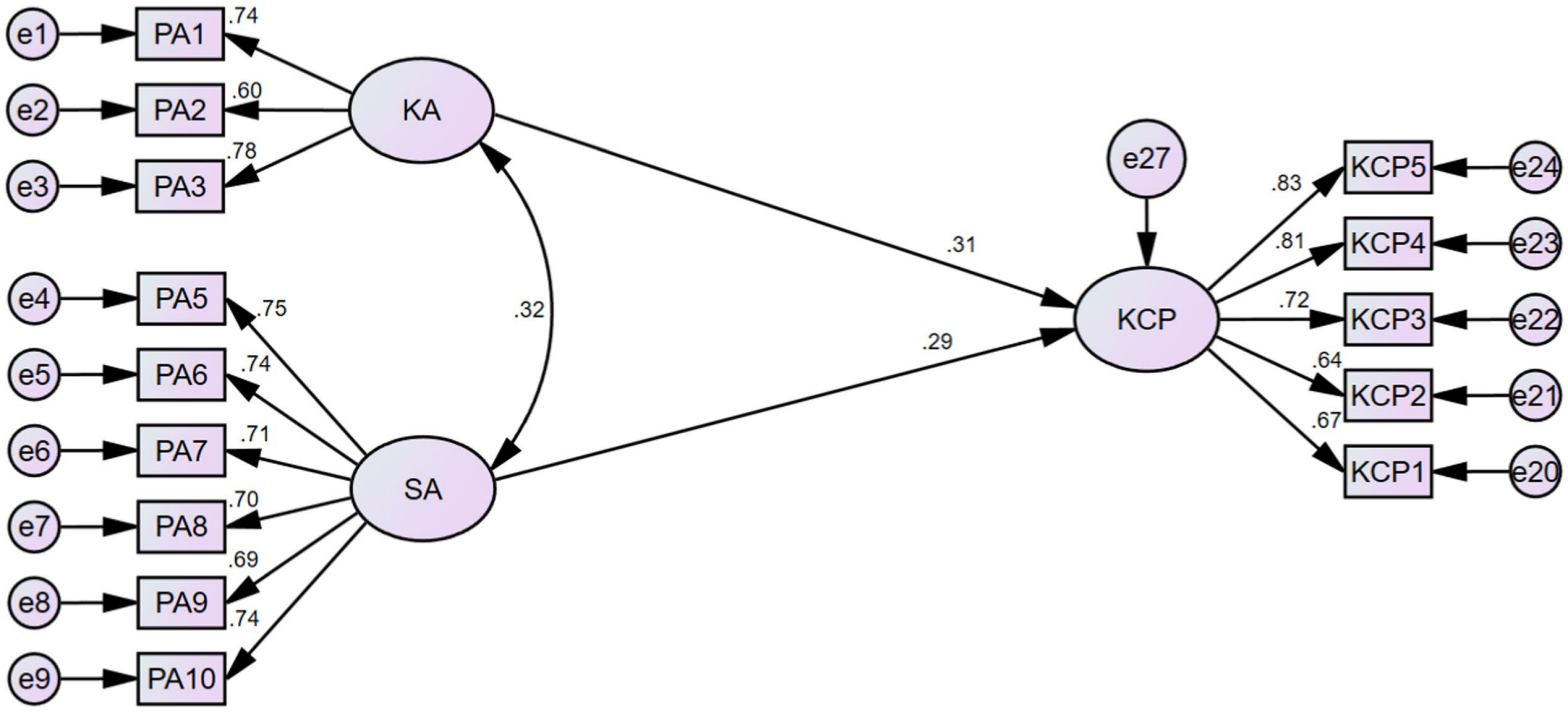
Structural equation modeling for the main effects.

Table 9 shows that the model fit statistics have reached the ideal value, which means that the main effects model is well-fitted. From Table 10, it can be seen that KA has a significant positive effect on KCP (*β* = 0.308, *p* < 0.001), and SA has a significant positive effect on KCP (*β* = 0.293, *p* < 0.001), so H1 and H2 are supported (Figure 2).

**Table 9:** The test for the main effects.

Measure	*χ* ^2^	*df*	*χ*^2^/*df*	RMSEA	SRMR	CFI	TLI	IFI
Threshold			<3.00	<0.08	<0.08	>0.9	>0.9	>0.9
Results	194.876	74	2.633	0.067	0.044	0.941	0.928	0.942

**Table 10:** Main effects path analysis.

Path	Standardized coefficient	SE	*t*-value	Value of *p*
KA → KCP	0.308	0.063	4.503	***
SA → KCP	0.293	0.062	4.597	***

***p < 0.001.

### Structural equation modeling for the mediating effects

Table 11 presents that the mediating effects model is well-fitted. In addition, the mediating effects path analysis shows that KA has a significant positive effect on CP (*β* = 0.327, *p* < 0.001); KA has a significant positive effect on EN (*β* = 0.245, *p* < 0.001); KA has no significant effect on SI (*β* = −0.036，*p* > 0.05). SA has no significant effect on CP (*β* = 0.023, *p* > 0.05); SA has no significant effect on EN (*β* = 0.083，*p* > 0.05); SA has a significant positive effect on SI (*β* = 0.325, *p* < 0.001); CP has a significant positive effect on KCP (*β* = 0.273, *p* < 0.001); EN has a significant positive effect on KCP (*β* = 0.382, *p* < 0.001); SI has a significant positive effect on KCP (*β* = 0.175, *p* < 0.01); KA has a significant positive effect on KCP (*β* = 0.165, *p* < 0.01); and SA has a significant positive effect on KCP (*β* = 0.212, *p* < 0.001). Therefore, except for KA to SI, SA to EN, and SA to CP, the hypotheses are all valid.

**Table 11:** The test for the mediating effects.

Measure	*χ* ^2^	*df*	*χ*^2^/*df*	RMSEA	SRMR	CFI	TLI	IFI
Threshold			<3.00	<0.08	<0.08	>0.9	>0.9	>0.9
Results	453.754	240	1.891	0.050	0.076	0.939	0.930	0.939

Previous studies (MacKinnon et al., 2004; Williams and MacKinnon, 2008) pointed out that the Bootstrap method is more statistically accurate than the causal steps approach and product of coefficient for testing indirect effects. One of the most significant advantages of the Bootstrap method is that the estimation of the indirect effect does not require the indirect effect to follow a normal distribution. Therefore, the Bootstrap method in this paper is used to test the mediating effect.

**Figure 3:**
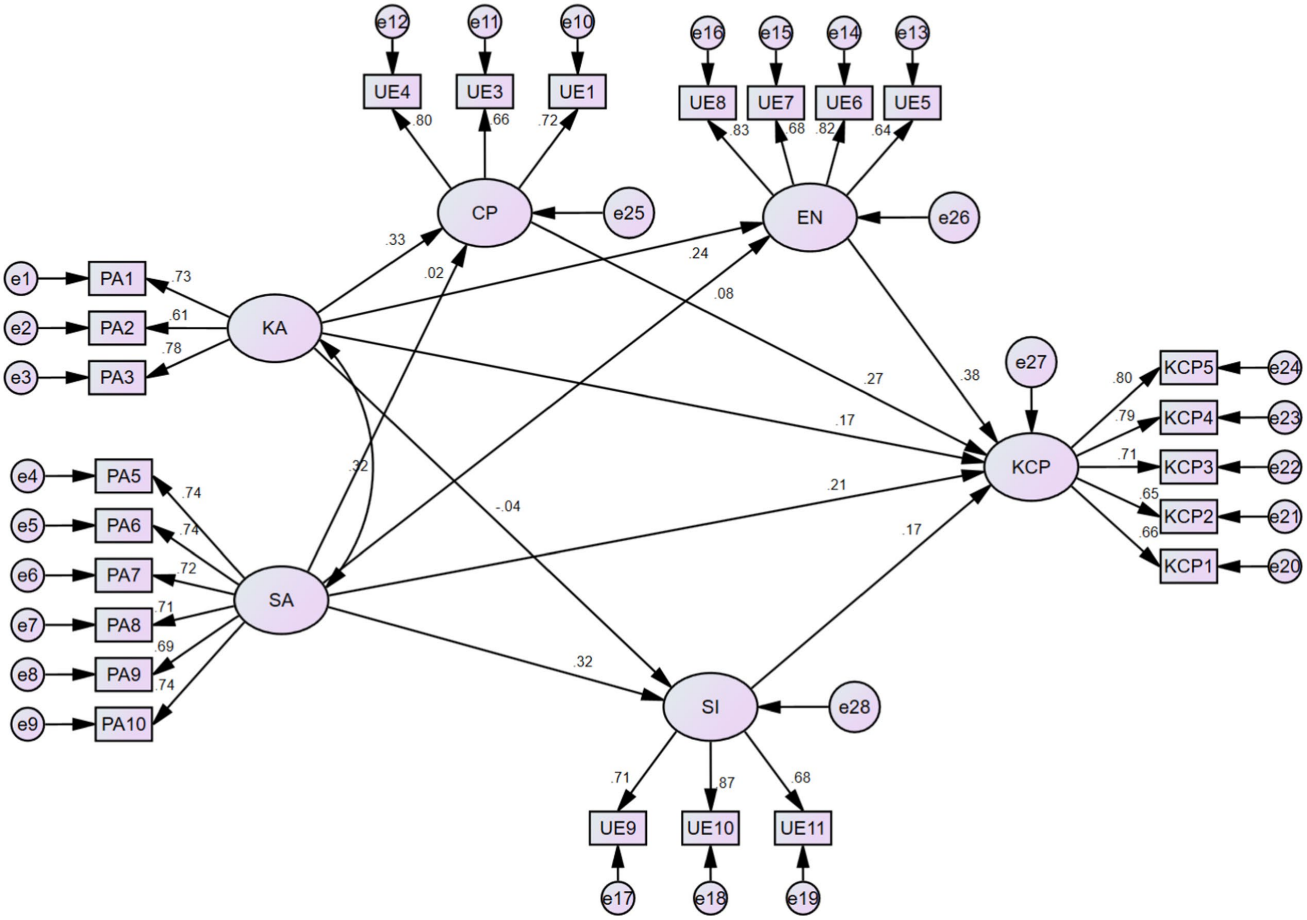
Structural equation modeling for the mediating effects.

From Table 12, we know that in the path of KA → CP → KCP, the confidence interval does not contain 0 (0.040, 0.164), so it shows that CP has a mediating effect between KA and KCP, and the size of the mediating effect is 0.089. In the path of KA → EN → KCP, the confidence interval does not contain 0 (0.039, 0.162), so it means that EN has a mediating effect between KA and KCP, and the size of the mediating effect is 0.094. In the path of KA → SI → KCP, the confidence interval contains 0 (−0.043, 0.023), thus indicating that SI does not have a mediating effect between KA and KCP. In the path of SA → CP → KCP, the confidence interval contains 0(−0.035, 0.047), so it means that there is no mediating effect of CP between SA and KCP. In the path of SA → EN → KCP, the confidence interval contains 0(−0.019, 0.093), thus indicating that EN does not have a mediating effect between SA and KCP. In the path of SA → SI → KCP, the confidence interval does not contain 0 (0.021, 0.117), thus indicating that SI plays a mediating role between SA and KCP, and the size of the mediating effect is 0.057 (Figure 3).

**Table 12:** Mediating effect analysis.

Path	Mediating effect	Bias-corrected 95% CI	
		Lower	Upper
KA → CP → KCP	0.089	0.040	0.164
KA → EN → KCP	0.094	0.039	0.162
KA → SI → KCP	−0.006	−0.043	0.023
SA → CP → KCP	0.006	−0.035	0.047
SA → EN → KCP	0.032	−0.019	0.093
SA → SI → KCP	0.057	0.021	0.117

Bootstrap = 5,000.

### Empirical results

According to the above empirical test results, most of the hypotheses in this paper are supported. Specifically, knowledge platform affordances influence knowledge collaboration performance positively, so H1 and H2 are supported. As a mediating role, user engagement also positively influences knowledge collaboration performance, so H9, H10, and H11 are supported. In terms of the relationship between knowledge platform affordances and user engagement, we can see that knowledge affordances have a positive effect on conscious participation and enthusiasm. However, they do not influence social interaction positively. Thus, H3 and H4 are supported, but H5 is rejected. Although social affordances have a positive effect on social interaction, neither conscious participation nor enthusiasm is positively related to social affordances. Thus, H6 and H7 are rejected but H8 is supported. From the mediating effect analysis, the results show that conscious participation and enthusiasm mediate the relation between knowledge affordances and knowledge collaboration performance. Additionally, social interaction mediates the relation between social affordances and knowledge collaboration performance. However, the paths of KA → SI → KCP, SA → CP → KCP, SA → EN → KCP are not proved. Therefore, H12a, H12b, and H12f are supported, but H12c, H12d, and H12e are rejected.

## Discussions

First, knowledge platform affordances have a direct and positive influence on knowledge collaboration performance. Knowledge affordances and social affordances can facilitate the realization of knowledge collaboration performance. This conclusion clarifies the relationship between affordances and knowledge collaboration performance and provides a basis for the research on the realization mechanism of knowledge collaboration performance.

Second, user engagement partially mediates the relation between knowledge platform affordances and knowledge collaboration performance. The results show that conscious participation and enthusiasm play a mediating role between knowledge affordances and knowledge collaboration performance, and social interaction mediates the relation between social affordances and knowledge collaboration performance. This complements the influencing factors of improving knowledge collaboration performance, focusing on the bi-directionality of user engagement from the perspective of user and platform interaction. Additionally, the application of the composition of user engagement proposed by Vivek (2009) in the knowledge platform is added. However, the paths of knowledge affordances-social interaction-knowledge collaboration performance, social affordances-conscious participation-knowledge collaboration performance, and social affordances-enthusiasm-knowledge collaboration performance are not supported. It can be seen that the two kinds of affordances of the knowledge platform can provide targeted promotion for user engagement. On the one hand, knowledge affordances enhance users’ enthusiasm and conscious participation. On the other hand, social affordances ensure the satisfaction of social psychological needs, thus forming a high degree of user stickiness. The reason of the result could be that users’ conscious participation and enthusiasm for engaging in the platform mainly come from the demand for knowledge resources themselves. Whereas the interactive needs of users to participate in the platform are motivated by the social functions provided by the platform. From the literature, we know that knowledge affordances reflect the user’s pursuit of knowledge resources from the knowledge platform, which is the interaction result between the user’s demand for certain knowledge and the platform’s knowledge resources (Shi et al., 2020). Since we decompose social interactions separately in both knowledge platform affordances and user engagement, this may cause the absence of cross effects between the two variables. This has also become a limitation of the research, and the configuration analysis may be the focus of the next research to determine whether there is a more complex causal relationship between knowledge platform affordances and user engagement.

Third, user engagement has a direct and positive influence on knowledge platform collaboration. Different from other studies that focus on the relationship between user engagement and behavioral outcomes like word of mouth and app rating (Wu et al., 2018; Bitrián et al., 2021), this paper puts the application scenario on the knowledge platform and focuses on how user engagement affects knowledge collaboration performance.

## Conclusions and implications

### Conclusion

In the context of the “sinking” of the Internet innovation market, how to accurately grasp the diverse psychological needs of a large number of non-traditional users and improve knowledge collaboration performance timely and effectively have become a core issue that knowledge platforms must face. Based on surveying users of Zhihu as objects, this paper uses the affordance theory to study the formation process of knowledge collaboration performance, revealing the mediating role of user engagement in the formation of performance, thus providing a theoretical basis for the operational decisions of digital content platforms.

By sorting and analyzing existing literature and related theories, this paper proposes a theoretical framework for the relationship among knowledge platform affordances, user engagement, and knowledge collaboration performance. In a nutshell, knowledge platform affordances can directly affect knowledge collaboration performance and act on it through user engagement. According to affordance theory, knowledge platform affordances are the set of possibilities for platform users to take behaviors using platform technology in a demand-oriented manner (Leonardi and Barley, 2010; Robey et al., 2012), which can be used to better understand the impact of a combination of new technologies and organizational characteristics on user behavior (Majchrzak and Faraj, 2013). It can be seen that the formation of platform knowledge collaboration performance is the result of the interaction of factors such as platform characteristics, user needs, and platform technology, which also means that platform decision-making must consider these three important factors at the same time, so as to avoid pure “traffic thinking.” Meanwhile, user engagement is an important dependency for the formation of platform performance, and the source of user engagement is also rooted in the high interaction of the above three elements. On top of this, it is necessary to pay attention to the interaction of the three to gain important user trust, user participation, and user stickiness.

Since the knowledge platform has the dual functions of seeking knowledge and socializing, the behavior of platform users naturally contains the needs of both aspects, thus forming two kinds of affordances of the platform: knowledge affordances and social affordances. The research in this paper finds that the conscious participation and enthusiasm of user engagement mainly come from the user’s pursuit of knowledge resources, while social interaction comes from social needs. This also shows that the knowledge platform is fundamental and critical in constructing high-quality knowledge resources, and the strengthening and improvement of the platform’s social function has an important synergistic and complementary role.

Moreover, it is manifested from the paper that user engagement of conscious participation, enthusiasm, and social interaction could enhance knowledge collaboration performance. Therefore, individuals who use knowledge platforms with intention, are passionate about knowledge platforms, or enjoy social interactions on knowledge platforms could contribute to the appreciation of knowledge capital and social capital.

### Implications

#### Theoretical implication

Open innovation with user engagement is an essential feature of digital platforms (Yu et al., 2018). Current research focuses on several aspects of user needs, user behavior, and platform performance. However, the formation mechanism of user engagement and performance is rarely explored from the perspective of the integration of the three. In the digital age, users have become common participants in the development of platforms (Zhang et al., 2021), while each platform has its unique affordances, which are the integrated products of users’ needs and platform features. As a result, it is important to understand the mechanism of knowledge collaboration performances based on platform affordances and user engagement.

Our study has enriched the existing studies by exploring deeper into the relationship among knowledge platform affordances, user engagement, and knowledge collaboration performance. It has verified the mediating value of user engagement between knowledge platform affordances and knowledge collaboration performance. Affordances provide a theoretical basis for revealing the underlying interaction mechanism of user engagement at the social-technical level. This topic firstly explores knowledge platform affordances and user engagement in the context of popular knowledge production and profoundly understands the matching of platform systems and users. Secondly, the realization of platform value can be explained by analyzing the relation between user engagement and knowledge collaboration performance.

#### Practical implication

In the fierce market competition, it is not easy to encourage users to engage in value co-creation (Wang and Jiang, 2018). User engagement is an effective way to enhance platform performance (Xia et al., 2021). In the context of this paper, the establishment of user engagement with knowledge platforms is an effective way of making knowledge platforms into a new generation of competitive marketing channels. Management of enterprises should be fully aware that knowledge platform affordances and user engagement play an important role in promoting platform value. It should be noticed that if the design of platforms could fully facilitate users to engage in platforms. Enterprises should pay attention to the differences in organizational characteristics determined by value positioning and resource endowments, and design more scientific and effective user engagement mechanisms at the socio-technical level, thereby promoting the high-quality development of the knowledge platforms. The research results of this topic have broad application prospects in knowledge platform enterprises and platform economy.

The current social production system is highly developed, with abundant commodities and diverse services. Each user has different consumption motivations and needs in different situations. For each platform, it is no longer simple “traffic thinking,” but it is necessary to match platform features with user needs and use digital technology to achieve this connection. The conclusions of this paper fully reveal that platforms in the digital economy era must pay attention to the matching of user needs and their own characteristics. Furthermore, it is necessary to carry out a more in-depth motivation and demand portrait of the platform user group, and conduct a comprehensive analysis of the platform positioning, value proposition, and core capabilities, so as to formulate the correct operation strategy.

## Limitations and future research

Due to the limitations of the author’s ability, research time, and the cost of questionnaires, this paper has certain limitations. The specific examples are shown as follows.

First, since this paper uses questionnaires to obtain data for empirical research, the type of data is cross-sectional data, which will cause the explanatory power of causal relationship inference between knowledge platform affordances, user engagement, and knowledge collaboration performance to be relatively decreased, so it cannot be completely determined that there is a clear causal relationship between these variables. Relevant studies recommend using different variables to obtain data at different time points for empirical testing. Therefore, it is necessary to further improve the data measurement procedures in the future and use time-series or longitudinal data to clarify the causal relationship between variables (Chen and Wu, 2011). In addition, research samples with a certain time span can better observe the impact of affordances and user engagement.

Second, the sample size of this paper is limited due to the author’s ability and time and cost constraints. The proportion of Zhihu users in first-and second-tier cities is relatively high. However, with the “sinking” of the Zhihu market, third-and fourth-tier users are gradually increasing, and female users have increased significantly. Therefore, the sample may lack certain universality, and a relatively large number of cities and populations should be further tested in the future.

Third, there may be joint complementary effects between the multiple affordances of the knowledge platform and the engagement ways, and configuration analysis of these variables should be carried out in the future. Through this analysis method, it is possible to reveal different affordance paths and engagement paths for obtaining high performance, not only to discover the differences in the effects of different affordances and engaging ways and their combinations, but also to discover the differences in the psychological needs of users. In this way, it could promote customer segmentation and user profiling and provide decision-making references for the efficient and healthy development of the platform.

In addition, although Zhihu is a successful enterprise, other knowledge platforms are worth studying. In the future, two or three knowledge platforms, such as Quora and Wukong Q&A, can be selected for comparative research. These platforms have different organizational characteristics (value positioning, resource endowment, and competitive strategy), but they are all question-and-answer knowledge platforms. There are both successful cases and failure cases, which is convenient for comparative research. In recent years, with the rapid development of the Internet knowledge industry, practical cases such as knowledge payment and free reading have emerged, and cases of value co-creation and co-destruction have emerged. Designing an effective user engagement mechanism for non-traditional partners still lacks mature theoretical guidance. The case study approach is also helpful for building theory and discovering new theoretical insights (Eisenhardt and Graebner, 2007).

## Data availability statement

The original contributions presented in the study are included in the article/supplementary material, further inquiries can be directed to the corresponding author.

## Author contributions

ZJ is responsible for the main structure and writing of the paper. JC is responsible for data collection and analysis. CL is responsible for the final changes and corrections. All authors contributed to the article and approved the submitted version.

## Conflict of interest

The authors declare that the research was conducted in the absence of any commercial or financial relationships that could be construed as a potential conflict of interest.

## Publisher’s note

All claims expressed in this article are solely those of the authors and do not necessarily represent those of their affiliated organizations, or those of the publisher, the editors and the reviewers. Any product that may be evaluated in this article, or claim that may be made by its manufacturer, is not guaranteed or endorsed by the publisher.

## Appendix 1 Questionnaire

Answers “1–7” express your opinion on the topic, and the score indicates a gradual progression from “strongly disagree” to “strongly agree.” Please select “√” according to your actual situation.

**Table:**

Knowledge platform affordances	Strongly disagree → strongly agree						
	1	2	3	4	5	6	7
1. I think the content on Zhihu is more accurate, detailed and convincing.							
2. I can Q&A for free or ask an expert for a low cost on Zhihu.							
3. I think Zhihu has rich content and many forms of content (videos, pictures, links to related knowledge points).							
4. I think there are many new ideas/concepts in Zhihu content.							
5. I can upload my own content on my personal homepage of Zhihu.							
6. I can share the content I am interested in with my friends on Zhihu.							
7. I can join online communities on Zhihu.							
8. I can comment on other’s posts on Zhihu.							
9. I can ask or answer questions on Zhihu.							
10. I can browse other people’s content on Zhihu.							
**User engagement**							
1. Anything related to Zhihu grabs my attention.							
2. I pay a lot of attention on Zhihu.							
3. I often browse content on Zhihu.							
4. When I encounter problems, I use Zhihu intentionally.							
5. I spend a lot of my discretionary time using Zhihu.							
6. My days would not be the same without using Zhihu.							
7. I am passionate about Zhihu.							
8. I am heavily into using Zhihu.							
9. I love discussing topics with users who have similar interests to me on Zhihu.							
10. I enjoy interacting with other Zhihu users.							
11. Using Zhihu is more fun when other people around me do it too.							
12. I often liking and commenting other Zhihu users’ content.							
**Knowledge collaboration performance**							
1. I learned new knowledge and skills from Zhihu.							
2. I can apply the knowledge I learned from Zhihu to solve practical problems.							
3. Interacting with other users on Zhihu broadens my horizons.							
4. I often share views or ideas with friends on Zhihu.							
5. I made new friends on Zhihu.							
